# A Review of Hyperglycemia in COVID-19

**DOI:** 10.7759/cureus.37487

**Published:** 2023-04-12

**Authors:** Maryam Zahedi, Saba Kordrostami, Mohammadreza Kalantarhormozi, Marziyeh Bagheri

**Affiliations:** 1 Department of Internal Medicine, Endocrinology, and Metabolism, Clinical Research Development Unit (CRDU) 5 Azar Hospital, Golestan University of Medical Sciences, Gorgan, IRN; 2 Department of Endocrinology and Diabetes, Clinical Research Development Unit (CRDU) 5 Azar Hospital, Golestan University of Medical Sciences, Gorgan, IRN; 3 Department of Endocrinology, Bushehr University of Medical Sciences, Bushehr, IRN; 4 Department of Internal Medicine, Bushehr University of Medical Sciences, Bushehr, IRN

**Keywords:** stress-induced hyperglycemia, hyperglycemia, prognosis, covid-19, diabetes mellitus

## Abstract

Diabetes mellitus (DM) is one of the most common chronic metabolic disorders worldwide, which increases the risk of common and opportunistic infections. Following the coronavirus disease 2019 (COVID-19) pandemic, a higher incidence rate, more severe forms of the disease, and exacerbation of hyperglycemia and its complications have been observed in patients with DM. Moreover, stress-induced hyperglycemia has been observed in many hospitalized nondiabetic patients after contracting COVID-19. Hyperglycemia worsens prognosis in both diabetic and nondiabetic patients. In this study, the mechanism of new-onset or exacerbation of hyperglycemia, the effect of the treatments used for COVID-19 on hyperglycemia, the importance and appropriate method of blood glucose (blood sugar (BS)) control during the disease, and the possible fate of new-onset hyperglycemia after recovery from COVID-19 to some extent is expressed.

## Introduction and background

According to the International Diabetes Federation (IDF), 537 million adults are estimated to be affected by diabetes mellitus (DM) in 2021, and by 2045, the prevalence of DM is expected to reach 783 million people. On the other hand, in 2021, the prevalence of DM in adults in Iran was estimated at around 15% [1].

There are various varieties of DM, with types 1 and 2 serving as its two primary subtypes. Type 1 DM (DM-I) occurs due to a complete or almost complete lack of insulin due to autoimmunity. In comparison, type 2 DM (DM-II), which includes more than 90% of DM cases, is initially characterized by different degrees of insulin resistance (IR), followed by a progressive decrease in insulin secretion. One of the leading causes of death and morbidity worldwide, DM is connected to a number of acute and chronic complications [2]. Hyperosmolar hyperglycemic state (HHS) and diabetic ketoacidosis (DKA) are two of the most significant acute consequences of DM. Diabetics who develop acute bacterial, viral, or fungal infectious illnesses, such as coronavirus disease 2019 (COVID-19), frequently experience DKA and HHS [3]. On the other hand, bacterial, viral, and rare opportunistic infections, including fungal infections, such as mucormycosis, are more common in diabetic patients, especially in those with uncontrolled blood glucose (blood sugar (BS)), due to cellular and humoral immune system dysfunction. In addition to the fact that diabetics are more prone to infection, the severity of the infection and its complications are significantly higher in these patients [4,5]. Studies show that the high BS level directly increases SARS-CoV-2 replication in human monocytes, and glycolysis allows the continuation of virus replication through mitochondrial reactive oxygen species (ROS). In other words, hyperglycemia can support the further multiplication of the virus in the body [6].

The World Health Organization (WHO) labeled COVID-19 a pandemic in March 2020. More than 102 million people contracted the virus by the end of January 2021. More than 2.2 million people have died as a result of COVID-19 worldwide, and the subsequent pandemic has greatly complicated global affairs [7]. Poor controlled DM, worsening of hyperglycemia in people with DM, and stress-induced hyperglycemia in nondiabetics are among the most common factors that cause the poor prognosis of COVID-19 [6,8,9].

Stress-induced hyperglycemia and insulin resistance (IR) during bed rest are caused by a number of variables, including medications (corticosteroids, vasopressors, and β-blockers), activation of inflammatory pathways, and the release of stress hormones. Other contributing factors are intravenous dextrose, antibiotic solutions, insufficient insulin secretion, and volume reduction. In addition, hospitalization (bed rest) induces IR by various mechanisms, even without any evident disease [10]. In infectious diseases, IR occurs with massive production of inflammatory cytokines, which disrupts insulin secretion [11].

Hyperglycemia causes inflammation and endothelial dysfunction [12] and an increase in the production of oxygen free radicals. According to evidence, these ROS are the cause of vascular endothelial damage in diabetics. Prolonged exposure to ROS in chronic hyperglycemia in diabetic patients increases antioxidant defense in cells, which ultimately protects the organ tissues to some extent [13,14]. However, this is not true in acute hyperglycemia in the absence of previous DM, and organ tissues are prone to more damage in these conditions [14]. Therefore, in hospitalized patients with COVID-19, stress-induced hyperglycemia seems to worsen the prognosis in nondiabetic patients even more than that of diabetics. Moreover, stress-induced hyperglycemia is a better predictor of outcome in COVID-19 patients than their pre-hospitalization glycemic status (as determined by hemoglobin A1c (HbA1c)) [15,16]. Since the mortality and severity of COVID-19 disease is higher by twofolds in the context of hyperglycemia and DM, identifying patients with stress-induced hyperglycemia and accurate control of BS levels can improve prognosis both in diabetics and nondiabetics [17].

## Review

Pathophysiology of COVID-19 and metabolic side effects

Acute respiratory distress syndrome (ARDS) is brought on by the pathogenic human COVID, which attaches to its target cells via angiotensin-converting enzyme 2 (ACE2) receptors. ACE2 is the primary hormone of the renin-angiotensin-aldosterone system (RAAS). The task of this system is to maintain water and sodium, as well as vascular resistance and thus blood pressure (BP) [6,9]. ACE2 exists in the bronchial epithelium, vascular endothelium, alveolar cells, kidney, and intestine [9,18]. This enzyme cleaves angiotensin (Ang) II and forms Ang 1-7 [18]. COVID-19 inhibits the ACE2 upon entering the cell, hence increasing the production of Ang II. Intact Ang II acts to stimulate inflammatory markers through the Ang I receptor (AT1R) and leads to aldosterone (Ald) release. Ald in turn increases blood pressure (BP) and causes hypokalemia, edema, bleeding, and ARDS by increasing vascular permeability. Therefore, COVID leads to acute lung damage [18,19]. Contrary to Ang II with pro-inflammatory effects, patients with COVID-19 benefit from the anti-inflammatory and anti-fibrotic properties of Ang 1-7, which helps them recuperate [18]. Also expressed in the pancreas, ACE2 is crucial for maintaining glucose homeostasis. Insulin secretion is reduced, and islet cells are damaged as a result of COVID binding to ACE2 [20]. Contrarily, the COVID virus alters glucose tolerance and raises BS by inducing IR and decreasing insulin release from the cells, which inhibits ACE2 and increases the synthesis of Ang II. Thus, it can be concluded that in patients with more severe COVID-19, the imbalance in the activity of AT1R and AT2R leads to metabolic disturbance, high BP, IR, and hyperglycemia [18].

ACE2 is highly expressed in the pancreas of diabetic patients. Due to the affinity of COVID-19 for ACE2, diabetics are more susceptible to infection with COVID-19. Moreover, DM raises the expression of ACE2 in the heart, lungs, and liver, which explains why it worsens multiorgan dysfunction in diabetics who have COVID-19 infections [6].

Insulin resistance and hyperglycemia in ICU patients

As was already established, stress-induced IR and increased glucose synthesis frequently result in hyperglycemia in critically ill patients. Independent of other circumstances, hyperglycemia increases mortality in patients hospitalized in the intensive care unit (ICU). Consequently, precise BS level management is crucial. Patients with recent hyperglycemia showed a three times greater mortality risk (31%) among cases admitted to the ICU compared to diabetic patients with controlled blood sugar (10%) or normoglycemic patients (11.3%). It is worth noting that the lowest mortality rate (9.6%) was seen in cases with BS between 80 and 99 mg/dL. The mortality rate rises as the patient’s BS level gradually increases to more than 300 mg/dL, reaching 42.5% [21].

Increased lipolysis, decreased muscle glucose absorption, and increased hepatic glucose synthesis are the hallmarks of IR. The liver, muscle, and fat tissues in this disease do not react to insulin. Hence, IR and hyperglycemia are linked to a number of risk factors for cardiovascular diseases, such as hypertension, dyslipidemia, inflammation, and glucose intolerance, as well as an aggravation of the COVID-19 illness [22]. Moreover, IR results in proteolysis, which elevates the danger of cardiovascular illness, acute renal failure, and demise [10].

According to several studies, hyperglycemia and IR happened shortly after lung disease but before ventilator-associated pneumonia was identified. Although the connection between lung disease and hyperglycemia is not fully understood, it is known that pancreatic cell failure rather than IR is the cause of hyperglycemia in patients [10]. Moreover, an increase in the level of glucose in the airways is brought on by hyperglycemia, which disturbs homeostasis, and inflammation of the lung epithelium. The proliferation of lung pathogens (viral, bacterial, etc.) is correlated with the level of pulmonary glucose and is inversely correlated with intracellular bactericidal activity. Hyperglycemia reduces leukocyte mobility and phagocytic activity. It eventually leads to endothelial dysfunction, apoptosis, and reduced lung antioxidants. The increased glucose levels in the airways, causing inflammation, also aggravate lung disease. Severe hyperglycemia also causes electrolyte imbalance and volume changes [23]. Previous studies showed that in the case of conditions with IR such as obesity, there is a possibility of developing DM and hypertension following critical illnesses. They also stated that hyperglycemia in ICU patients is nonphysiological [10]. Hyperglycemia is known to be a potent pro-inflammatory mediator. It has been demonstrated that maintaining a blood sugar level of 110 mg/dL or below protects against death and morbidity. Healthy subjects receive 75 g of oral glucose, which raises inflammatory markers and ROS generation. Nuclear factor kappa B (NF-kB), which has thrombotic characteristics and raises ROS, is activated by hyperglycemia [24].

Pneumonia is known as one of the most important causes of death caused by COVID-19 infection. Patients with diabetes are more likely to contract infections, particularly the flu and pneumonia. The clearance of microorganisms from the lung is lessened by DM, as was mentioned in the context of hyperglycemia, and granulocyte cells’ capacity to phagocytose is also diminished. Controlled DM improperly promotes bacterial growth. For a variety of reasons, including increased exposure to ROS, leukocyte malfunction, lower lung elasticity, muscular weakness, inflammatory induction, and increased glycosylation of proteins, hyperglycemia can generally affect lung function (e.g., advanced end-product glycosylation). Lungs are elastic, but hyperglycemia reduces lung elasticity. Lungs are exposed to ROS the most. On the other hand, DM causes pulmonary microangiopathy and disruption of oxygen exchange [23].

Possible adverse effects of bed rest on hyperglycemia in COVID-19 patients

The adverse effects of prolonged bed rest on the musculoskeletal system have already been documented [25]. In a study of 20,515 patients on bed rest, pressure ulcers were found in 527 (2.57%) people, deep vein thrombosis in 343 (1.67%), pneumonia in 1,647 (8.16%), and urinary tract infection in 265 (1.29%) [26]. An increase in BS and IR may occur even with less than a week of bed rest [27]. Meanwhile, many adverse effects of hyperglycemia are known in infection, impaired wound healing, cardiovascular diseases, myocardial infarction, atrial fibrillation, pericarditis, cerebral ischemia, and respiratory and neurological complications [28].

Also, decreased physical activity, which is common among patients admitted to the ICU, can cause an increase in BS and IR, while stress-induced hyperglycemia during an ICU stay, caused by multiple factors, including bed rest and inactivity, is associated with an increased risk of DM [29]. IR ensues even when energy balance persists during periods of immobility during hospitalization and ICU admission [30]. Hyperglycemia and the disruption of glucose homeostasis in hospitalized patients are often multifactorial. Changes in the metabolism of glucose and lipids, the release of stress hormones, generated ROS, and inflammation are some of these elements [31]. Hence, a deeper comprehension of the workings of bed rest in COVID-19 patients is required, especially in light of the prevalence and mortality rate.

Stress level is high in COVID-19 patients. Stress causes hypothalamus-pituitary-adrenal (HPA) axis stimulation in some acute diseases. The production of stress hormones such as catecholamines, cortisol, growth hormone (GH), and glucagon is the key factors in general adaptability to acute illnesses. When people are stressed, the blood content of these hormones can rise by 2-5 times. A suitable and adaptive reaction known as the acute response to critical illness takes place in the initial days following the onset of the illness. Stress-induced mild to moderate hyperglycemia has a protective effect because it provides the brain and immune system with energy. However, numerous stress-related hormonal reactions result in long-lasting hyperglycemia and IR, which can be seriously harmful over time [31]. The mentioned stress hormones increase gluconeogenesis and glycogenolysis. Glucagon causes gluconeogenesis more than epinephrine and cortisol. The tumor necrosis factor-α (TNF-α) also increases gluconeogenesis by stimulating more glucagon production. However, glycogenolysis occurs firstly under the influence of catecholamines, followed by epinephrine and cortisol. On the other hand, epinephrine can induce IR by suppressing glucose uptake by skeletal muscles [10]. Tyrosine kinase activity is inhibited by catecholamines, which also inhibit glucose transporter type 4 (GLUT-4). GH decreases insulin receptors and blocks tyrosine kinase’s inhibitory function [24,32]. Similarly, stress-related hormone production can trigger lipolysis, raise levels of free fatty acids (FFAs), and prevent peripheral tissues from absorbing glucose. Contrarily, despite hyperinsulinemia, the rise in FFAs also reduces insulin sensitivity and blocks the insulin signal in patients with severe illnesses. Increased superoxide generation, mitochondrial dysfunction, and impaired antioxidant enzyme activity are further effects of elevated FFA levels. Contrarily, cytokines such as TNF-α and interleukin-1 (IL-1) block insulin’s post-receptoral signal. The release of cytokines and IR is increased by the severity of the disease [10]. All these events cause IR and hyperglycemia, ultimately increasing mortality [24,32].

Inflammation in COVID-19 patients and its relationship with DM and hyperglycemia

Patients with COVID-19 experience large increases in inflammatory cytokines and C-reactive protein (CRP) [21]. A mixture of inflammatory immunoactive chemicals, including interleukins, interferons, chemokines, and TNF, are responsible for this inflammatory response [6]. Organ failure occurs in COVID-19 patients who are critically unwell as a result of cytokine storm (significant rise in inflammatory cytokines). Cytokines, as was previously said, can result in IR. TNF-α production is elevated in COVID-19 patients, which disrupts the insulin signaling pathway [33]. During an ICU hospital stay, interleukin-6 (IL-6) levels rise [10]. Also, during bed rest, several organs produce more nuclear factor kappa-light-chain-enhancer of activated B cells (NF-B) and toll-like receptor 4 (TLR4), and these inflammatory markers are linked to insulin resistance and hyperglycemia [32]. The inflammatory response reduces GLUT-4 turnover, leading to cellular glucose uptake inhibition, resulting in IR and hyperglycemia [32,34]. Also, pulmonary disease causes inflammation and cytokine response, resulting in hyperglycemia [10]. In pulmonary diseases, systemic inflammation and increased TNF-α and interleukin-5 (IL-5) cause hyperglycemia with impaired skeletal muscle glucose absorption [34]. Reduced insulin function is linked to increased inflammation severity in lung illnesses [10]. The likelihood of poor outcomes is increased by this hyperglycemia, especially in ICU patients with lung injury [11]. However, the combination of DM and COVID-19 infection dramatically increases inflammation, which in turn raises the mortality rate [9].

COVID-19-associated mucormycosis

Mucormycosis is a fatal fungal infection caused by several organisms of the genera *Rhizopus*, *Mucor*, and *Lichtheimia* [4]. Few studies have reported COVID-19-associated mucormycosis (CAM). This concurrent fungal infection was attributed to the immunocompromised system, which, in addition to the comorbidities such as DM, results in the reduction of WBC levels and other immune factors in affected patients. This in turn leads to cytokine storm and cellular organ impairment. Those who contracted COVID-19 in its second wave also had to deal with opportunistic illnesses brought on by bacteria and fungus. Although mucormycosis has been known to exist for many years, it was only recently discovered to be common among COVID-19 patients. Immunocompromised people are more likely to develop mucormycosis when they breathe in filamentous fungus from the environment or through COVID-19 care facilities. Due to being immunocompromised, COVID-19 patients with DM were more likely to also have mucormycosis. Despite its dire consequences and numerous case reports, mucormycosis is still neglected and understudied [5]. Uncontrolled BS and the corticosteroids used to control the inflammation are the major predisposing factors to CAM. Moreover, oxygen masks for managing critically and very ill patients with COVID-19 fostered opportunistic infections including mucormycosis [5,35-37]. Other factors such as increased ion levels have been also investigated, which needs further studies [35]. The CAM mortality rate reaches 29%, and 17% of affected patients are at risk of losing their eyes. Hence, a high index of suspicion and a multidisciplinary approach, including antifungal medication and surgical treatment, are crucial for a better outcome [38].

Effect of COVID-19 drugs on hyperglycemia

Systemic corticosteroids are usually used in the treatment of COVID-19 patients who need oxygen therapy [39]. We already know that systemic corticosteroids cause hyperglycemia, especially postprandial glucose, with the dysfunction of pancreatic β-cells, causing IR, which raises the need for insulin therapy [6]. Although due to the corticosteroids’ vital anti-inflammatory effects, there is no other choice but to continue using them in necessary cases, certainly with a controlled dose and duration [39].

Protease inhibitors, such as lopinavir and ritonavir, from the category of antiviral drugs, are used in the past in the treatment of patients with HIV infection. These drugs may cause hyperglycemia, new-onset DM, or exacerbation of hyperglycemia in people with a history of pre-DM or diabetic ketoacidosis in diabetic patients. Insulin sensitivity and β-cell function can be impaired by 50% in patients with HIV infection under treatment with these drugs [6].

The FDA has approved remdesivir, an antiviral medication having an RNA nucleotide inhibitory mode of action, for the treatment of COVID-19 patients who need oxygen. Remdesivir may result in hyperglycemia [39]. In mice given a high-fat diet, remdesivir reduced hyperglycemia, insulin resistance, and fatty liver. An increase in blood glucose level was evaluated in the group treated with remdesivir and the placebo group in two multicenter clinical trial studies and in Chinese patients. There is still a need for more evidence to measure its effect on glucose metabolism [6].

Ivermectin is an antiparasitic drug used in the treatment of COVID-19. Although existing studies are not enough to prove its effectiveness in treating COVID-19, some investigations showed that ivermectin can cause hyperglycemia [39].

Chloroquine and hydroxychloroquine are anti-inflammatory and antimalarial drugs that are sometimes used in the treatment of COVID-19. Although their effectiveness has not been proven, these drugs do not only cause hyperglycemia but also lower BS [39]. Hydroxychloroquine prevents the binding of COVID-19 to the AT2R. Hydroxychloroquine reduces glucose levels by improving insulin sensitivity and β-cell function [6]. Hence, these medications can be utilized to treat COVID-19-positive diabetic patients [39].

Azithromycin is another drug that has sometimes been used in the treatment of COVID-19 for some reason, although it did not show any benefit. Currently, there is not enough information about its effect on BS levels [39].

Biological anti-cytokine treatments have been used in very severe cases of COVID-19 [40]. Among these drugs, IL-6 receptor inhibitors are used in the treatment of very severe cases of COVID-19 with lung damage and high levels of IL-6. They also showed benefits in glucose tolerance and IR in patients with rheumatoid arthritis (RA). Anakinra, an interleukin-1 (IL-1) inhibitor drug that significantly improves respiratory function in patients with severe COVID-19, improves BS and β-cell function in diabetic patients. On the contrary, canakinumab, another IL-1-β inhibitor drug that is under investigation for the possibility of its use in the treatment of COVID-19, is not effective in the treatment of newly diagnosed DM-I [6]. The TNF inhibitor, adalimumab, has also been used for the inflammatory phase of COVID-19. This class of drugs showed improvement in hyperglycemia, IR, and β-cell function in patients with active RA [6].

In general, there is no sufficient information on the effect of biological treatments such as monoclonal antibodies on the BS of patients with COVID-19 [39]. Following the injection of most types of anti-COVID-19 vaccines, a mild and transient increase in BS has been reported between one and seven days [39]. In summary, among anti-COVID-19 treatments, corticosteroids and possibly antiviral treatments and vaccinations can cause hyperglycemia [39].

Treatment of COVID-19 patients with hyperglycemia

There are few studies on treating or preventing complications of COVID-19 patients admitted to the ICU. In addition to the conventional COVID-19 treatments, aggressive hyperglycemia control with insulin therapy can lessen some side effects in patients who have been on mechanical breathing for a week or more [21,31]. As was already established, hyperglycemia increases inflammation and increases the risk of oxidative stress (OS), pneumonia, and death. Previous studies prove that strict glucose control with insulin to less than 110 mg/dL in ICU patients is associated with anti-inflammatory effects, reducing complications and mortality [41].

Control of hyperglycemia, inflammation, and local changes in respiratory glucose homeostasis should be taken into account when treating patients hospitalized in the intensive care unit (ICU) [23]. The target BS concentration for patients hospitalized in the ICU with glucose levels between 140 and 180 mg/dL has been raised by the 2022 American Diabetes Association (ADA) guideline [41]. For patients in serious condition, insulin infusion is advised. The risk of pneumonia, multiple organ failure, and inflammation, the requirement for mechanical ventilation, and ICU dependence is decreased with accurate insulin hyperglycemia control [23]. It is recommended to check BS every one or two hours until the insulin infusion (short-acting) rate is adjusted. After the stabilization of BS levels, measurements should change at four-hour intervals [42].

Several methods are used by dipeptidyl peptidase-4 (DPP4) inhibitors and glucagon-like peptide-1 (GLP-1) analogs to lower hyperglycemia. This class of medications works by boosting insulin production, delaying stomach emptying, and reducing postprandial glucagon in a glucose-dependent manner. It is safe and efficient to use DPP4 inhibitors alone or in conjunction with insulin to regulate blood sugar levels without raising the risk of hypoglycemia or excessive weight gain [43]. In animal models, DPP4 inhibitors have anti-inflammatory actions and lower ACE levels. They might therefore be helpful for COVID-19-positive diabetic patients, despite the fact that there is currently little information about the effectiveness of DPP4 inhibitors in treating hyperglycemia in COVID-19 patients [44]. Also, all diabetic patients with COVID-19 treated with GLP-1 analogs should be carefully monitored and given adequate fluids and regular meals to avoid the risk of dehydration [6]. The elevation of ACE2 in the kidneys caused by sodium-glucose cotransporter-2 (SGLT-2) inhibitors may result in tissue damage [43]. As a result, SGLT-2 inhibitors are not advised for use in patients with COVID-19 due to the possibility of negative side effects [6].

Sulfonylurea may result in hypoglycemia in acute disorders (such as COVID-19 infection) due to low-calorie intake. Hence, especially in ICU patients, this medication group is not optimal for reducing high BS levels of COVID-19 [43].

By reducing the binding capacity, metformin can prevent COVID-19 from entering the cell. Moreover, metformin has anti-inflammatory and antioxidant properties. As a result, it is advantageous for individuals with DM who are hospitalized for COVID-19 but do not have liver or renal failure. Lactic acidosis risk is increased because kidney illness causes metformin to build up, and liver disease decreases lactate excretion. Due to the risk of lactic acidosis, metformin should be administered with caution in critically ill patients despite its benefits [6].

Thiazolidinediones can cause edema, weight gain, increased heart failure, and fluid retention. Therefore, these agents are inappropriate in patients with DM and COVID-19 [43].

Insulin should be the main treatment in the case of stress-induced hyperglycemia. Most of the available data did not show any difference in the types of DM in the affected patients [6].

Clinical course of newly diagnosed hyperglycemia in COVID-19 patients

Newly diagnosed hyperglycemia following viral infection has been one of the important disorders associated with the COVID-19 pandemic. However, it is unclear whether it is actually the manifestation of a previously unknown condition that could potentially lead to the worsening of pre-DM or new-onset DM caused by COVID-19. This sort of hyperglycemia tends to have unusual manifestations, including severe and resistant hyperglycemia and life-threatening DKA. The clinical course of hyperglycemia varies in terms of duration and outcome. In many people, emerging DM recovers completely or gradually after recovery from COVID-19. However, people who survive COVID-19 have been shown to have up to a 40% higher risk of developing DM in the first year, even in not very severe cases of COVID-19. Autoimmune damage caused by viral infection may also lead to type 1 DM. On the other hand, there may be an increase in the incidence of DM in the next wave after the epidemic [45].

Lastly, a continuing follow-up of patients with hyperglycemia is essential, especially for those with stress-induced hyperglycemia and new-onset DM during COVID-19. Long-term surveillance will improve early diagnosis, treatment, and follow-up of patients who are at risk of developing DM. Patients who remain diabetic will benefit from regular monitoring of risk factors for cardiovascular and kidney damage to reduce the possibility of future microvascular and macrovascular complications [46].

## Conclusions

The studies reviewed in this article state that the possibility of contracting more severe types of infection and complications of COVID-19 in case of simultaneous hyperglycemia is more in diabetic patients and in acute hyperglycemia. On the other hand, the infection itself and the treatments used cause hyperglycemia. Considering the high mortality rate in hyperglycemia, correct and timely control of BS and proper treatment of these patients are very important, which leads to a better prognosis. Finally, long-term follow-up of COVID-19 patients with hyperglycemia is also needed, as nondiabetic patients with stress-induced hyperglycemia are at higher risk of developing DM in the future.

## References

[1] Hariri S, Rahimi Z, Hashemi-Madani N (2021). Prevalence and determinants of diabetes and prediabetes in southwestern Iran: the Khuzestan comprehensive health study (KCHS). BMC Endocr Disord.

[2] van Wilpe R, Hulst AH, Siegelaar SE, DeVries JH, Preckel B, Hermanides J (2023). Type 1 and other types of diabetes mellitus in the perioperative period. What the anaesthetist should know. J Clin Anesth.

[3] Kim NY, Ha E, Moon JS, Lee YH, Choi EY (2020). Acute hyperglycemic crises with coronavirus disease-19: case reports. Diabetes Metab J.

[4] Islam MR, Rahman MM, Ahasan MT (2022). The impact of mucormycosis (black fungus) on SARS-CoV-2-infected patients: at a glance. Environ Sci Pollut Res Int.

[5] Azhar A, Khan WH, Khan PA, Alhosaini K, Owais M, Ahmad A (2022). Mucormycosis and COVID-19 pandemic: clinical and diagnostic approach. J Infect Public Health.

[6] Lim S, Bae JH, Kwon HS, Nauck MA (2021). COVID-19 and diabetes mellitus: from pathophysiology to clinical management. Nat Rev Endocrinol.

[7] Taylor JW, Taylor KS (2023). Combining probabilistic forecasts of COVID-19 mortality in the United States. Eur J Oper Res.

[8] Zhou W, Ye S, Wang W, Li S, Hu Q (2020). Clinical features of COVID-19 patients with diabetes and secondary hyperglycemia. Diabetes Metab J.

[9] Fang L, Karakiulakis G, Roth M (2020). Are patients with hypertension and diabetes mellitus at increased risk for COVID-19 infection?. Lancet Respir Med.

[10] Hsu CW (2012). Glycemic control in critically ill patients. World J Crit Care Med.

[11] Ceriello A, De Nigris V, Prattichizzo F (2020). Why is hyperglycaemia worsening COVID-19 and its prognosis?. Diabetes Obes Metab.

[12] Gero D (2017). Hyperglycemia-induced endothelial dysfunction. Endothelial dysfunction - old concepts and new challenges.

[13] Ceriello A, dello Russo P, Amstad P, Cerutti P (1996). High glucose induces antioxidant enzymes in human endothelial cells in culture. Evidence linking hyperglycemia and oxidative stress. Diabetes.

[14] La Sala L, Mrakic-Sposta S, Micheloni S, Prattichizzo F, Ceriello A (2018). Glucose-sensing microRNA-21 disrupts ROS homeostasis and impairs antioxidant responses in cellular glucose variability. Cardiovasc Diabetol.

[15] Mondal S, DasGupta R, Lodh M (2022). Stress hyperglycemia ratio, rather than admission blood glucose, predicts in-hospital mortality and adverse outcomes in moderate-to severe COVID-19 patients, irrespective of pre-existing glycemic status. Diabetes Res Clin Pract.

[16] Singh AK, Singh R (2020). Hyperglycemia without diabetes and new-onset diabetes are both associated with poorer outcomes in COVID-19. Diabetes Res Clin Pract.

[17] Kumar A, Arora A, Sharma P (2020). Is diabetes mellitus associated with mortality and severity of COVID-19? A meta-analysis. Diabetes Metab Syndr.

[18] Bornstein SR, Dalan R, Hopkins D, Mingrone G, Boehm BO (2020). Endocrine and metabolic link to coronavirus infection. Nat Rev Endocrinol.

[19] Zhang L, Liu Y (2020). Potential interventions for novel coronavirus in China: a systematic review. J Med Virol.

[20] Lu CL, Wang Y, Yuan L, Li Y, Li XY (2014). The angiotensin-converting enzyme 2/angiotensin (1-7)/Mas axis protects the function of pancreatic β cells by improving the function of islet microvascular endothelial cells. Int J Mol Med.

[21] Pakhetra R, Garg MK, Suryanarayana KM (2011). Management of hyperglycemia in critical illness: review of targets and strategies. Med J Armed Forces India.

[22] Govender N, Khaliq OP, Moodley J, Naicker T (2021). Insulin resistance in COVID-19 and diabetes. Prim Care Diabetes.

[23] Abbasi-Oshaghi E, Mirzaei F, Khodadadi I (2021). Letter to the editor regarding 'COVID-19 and diabetes: what does the clinician need to know?'. Prim Care Diabetes.

[24] Marik PE, Raghavan M (2004). Stress-hyperglycemia, insulin and immunomodulation in sepsis. Intensive Care Med.

[25] Sagarra-Romero L, Viñas-Barros A (2020). COVID-19: short and long-term effects of hospitalization on muscular weakness in the elderly. Int J Environ Res Public Health.

[26] Wu X, Li Z, Cao J (2018). The association between major complications of immobility during hospitalization and quality of life among bedridden patients: a 3 month prospective multi-center study. PLoS One.

[27] Larsen S, Lundby AM, Dandanell S (2018). Four days of bed rest increases intrinsic mitochondrial respiratory capacity in young healthy males. Physiol Rep.

[28] Farahani F, Mirzaei F, Khodadadi I, Abbasi-Oshaghi E (2020). Importance of hyperglycemia in preoperative, intraoperative and postoperative periods in COVID-19 patients. Int J Surg.

[29] Ali Abdelhamid Y, Kar P, Finnis ME (2016). Stress hyperglycaemia in critically ill patients and the subsequent risk of diabetes: a systematic review and meta-analysis. Crit Care.

[30] Truong AD, Fan E, Brower RG, Needham DM (2009). Bench-to-bedside review: mobilizing patients in the intensive care unit--from pathophysiology to clinical trials. Crit Care.

[31] Xiu F, Stanojcic M, Diao L, Jeschke MG (2014). Stress hyperglycemia, insulin treatment, and innate immune cells. Int J Endocrinol.

[32] Cyphert TJ, Morris RT, House LM (2015). NF-κB-dependent airway inflammation triggers systemic insulin resistance. Am J Physiol Regul Integr Comp Physiol.

[33] Marik PE, Bellomo R (2013). Stress hyperglycemia: an essential survival response!. Crit Care.

[34] Austin RL, Rune A, Bouzakri K, Zierath JR, Krook A (2008). siRNA-mediated reduction of inhibitor of nuclear factor-kappaB kinase prevents tumor necrosis factor-alpha-induced insulin resistance in human skeletal muscle. Diabetes.

[35] Sharma R, Kumar P, Rauf A (2022). Mucormycosis in the COVID-19 environment: a multifaceted complication. Front Cell Infect Microbiol.

[36] Al-Tawfiq JA, Alhumaid S, Alshukairi AN (2021). COVID-19 and mucormycosis superinfection: the perfect storm. Infection.

[37] Samaranayake LP, Fakhruddin KS, Ngo HC, Bandara HM, Leung YY (2022). Orofacial mycoses in coronavirus disease-2019 (COVID-19): a systematic review. Int Dent J.

[38] Watanabe A, So M, Mitaka H (2022). Clinical features and mortality of COVID-19-associated mucormycosis: a systematic review and meta-analysis. Mycopathologia.

[39] Parise R, Deruiter J, Ren J (2022). Impact of COVID-19 therapy on hyperglycemia. Diab Vasc Dis Res.

[40] Fana SE, Esmaeili F, Esmaeili S, Bandaryan F, Esfahani EN, Amoli MM, Razi F (2021). Knowledge discovery in genetics of diabetes in Iran, a roadmap for future researches. J Diabetes Metab Disord.

[41] Bar-Or D, Rael LT, Madayag RM (2019). Stress hyperglycemia in critically ill patients: insight into possible molecular pathways. Front Med (Lausanne).

[42] Pérez-Calatayud ÁA, Guillén-Vidaña A, Fraire-Félix IS, Anica-Malagón ED, Briones Garduño JC, Carrillo-Esper R (2017). [Metabolic control in the critically ill patient an update: hyperglycemia, glucose variability hypoglycemia and relative hypoglycemia]. Cir Cir.

[43] Gomez-Peralta F, Abreu C, Gomez-Rodriguez S, Barranco RJ, Umpierrez GE (2018). Safety and efficacy of DPP4 inhibitor and basal insulin in type 2 diabetes: an updated review and challenging clinical scenarios. Diabetes Ther.

[44] Sebastián-Martín A, Sánchez BG, Mora-Rodríguez JM, Bort A, Díaz-Laviada I (2022). Role of dipeptidyl peptidase-4 (DPP4) on COVID-19 physiopathology. Biomedicines.

[45] Pergolizzi J, LeQuang JA, Breve F, Magnusson PM, Varrassi G (2023). Exploring the implications of new-onset diabetes in COVID-19: a narrative review. Cureus.

[46] Khunti K, Del Prato S, Mathieu C, Kahn SE, Gabbay RA, Buse JB (2021). COVID-19, hyperglycemia, and new-onset diabetes. Diabetes Care.

